# Comparison of subsequent infection in methicillin-resistant *Staphylococcus aureus* nasal carriers between ST72 community-genotype and hospital genotypes: a retrospective cohort study

**DOI:** 10.1186/s13756-017-0220-1

**Published:** 2017-06-12

**Authors:** So Yeon Park, Doo Ryeon Chung, Yu Ri Kang, So Hyun Kim, Sun Young Cho, Young Eun Ha, Cheol-In Kang, Kyong Ran Peck, Jae-Hoon Song

**Affiliations:** 1 Division of Infectious Diseases, Department of Internal Medicine, Samsung Medical Center, Sungkyunkwan University School of Medicine, 81 Irwon-ro, Gangnam-gu, Seoul, 06351 Republic of Korea; 2 Center for Infection Prevention and Control, Samsung Medical Center, Seoul, South Korea; 3 Asia Pacific Foundation for Infectious Diseases, Seoul, South Korea; 4 Present address: Division of Infectious Diseases, Kangdong Sacred Heart Hospital, Hallym University College of Medicine, Seoul, South Korea

**Keywords:** Genotype, Kaplan-meier estimate, Methicillin-resistant *Staphylococcus aureus*, Cohort study, Carrier state

## Abstract

**Background:**

Carriage of methicillin-resistant *Staphylococcus aureus* (MRSA) is an important risk factor of subsequent infection. The purpose of our study was to compare the rates of subsequent infection among newly-admitted patients carrying MRSA between community-genotype and hospital-genotypes

**Methods:**

In this retrospective cohort study, we compared the rates of subsequent MRSA infection, time to subsequent infection and mortality in the following 6 months between the community-genotype ST72 MRSA cohort and the hospital-genotypes ST5 / ST239 MRSA cohort.

**Results:**

We identified 198 patients carrying ST72 and 156 patients carrying ST5 or ST239. There was no difference in the rates of subsequent infection between ST72 cohort and ST5 / ST239 cohort (13.1% vs. 12.8%; *P* = 0.931). The median time to development of subsequent infection was not significantly different (27 days vs. 88 days; *P* = 0.0877). The Kaplan-Meier method showed no difference in the cumulative rate of being free of subsequent infection between the cohorts (*P* = 0.9209). Overall mortality rates at 6 months did not differ (1.5% vs. 1.9%; *P* = 1.000)

**Conclusions:**

We found no evidence that rates of subsequent MRSA infection were different between newly-admitted patients carrying community-genotype ST72 MRSA and those whom carrying hospital-genotypes ST5 or ST239 MRSA.

## Background

Methicillin-resistant *Staphylococcus aureus* (MRSA) infections have imposed a high burden on healthcare resources as well as significant morbidity and mortality, and a recent emergence of community-associated MRSA (CA-MRSA) worldwide has added another concern. MRSA carriage has been identified as a strong risk factor for subsequent infection [1, 2]. Approximately 11% - 25% of patients who newly acquired MRSA were reported to develop subsequent infection during hospitalization [3, 4]. Although several CA-MRSA clones have emerged as important pathogens both in the community and the hospitals worldwide [5–7] and have shown different characteristics in the pattern of colonization and infection from the hospital-genotype MRSA clones [7, 8], there has been no study comparing the rates of subsequent MRSA infection in hospitalized patients carrying MRSA between the community-genotype and hospital-genotype strains.

In Korea, dominant MRSA clones endemic in the hospitals have been sequence type 5 (ST5) and ST239 clones, while ST72 clone has emerged in the community and has spread into the hospitals [9–11]. A recent single center study from Korea showed that the most frequent genotype in newly-admitted patients carrying MRSA was ST72, followed by ST5 and ST239 [12]. In this study, we compared the rates of subsequent MRSA infection among newly-detected hospitalized patients carrying MRSA between the community-genotype and the hospital-genotype cohort.

## Methods

### Study design and study participants

A retrospective cohort study was performed at Samsung Medical Center (Seoul, Republic of Korea), a university-affiliated tertiary care hospital with 1983 patient beds. We identified patients who were newly-detected MRSA nasal carriers between January 1, 2007 and March 31, 2010 from the previous study [12]. MRSA nasal screening was performed in selected newly-admitted patients according to the active surveillance policy for infection prevention purpose of the hospital. The indications for screening included the referral patients from the other hospitals or the chronic care facilities, the patients scheduled for operations in the departments of orthopedic surgery, neurosurgery, thoracic surgery and general surgery, the patients receiving hemodialysis or peritoneal dialysis, MRSA isolation from clinical specimens and carriage of vancomycin-resistant *Enterococcus* (VRE). Conventional culture methods were used for screening and confirmation of nasal carrier status of MRSA. The MRSA isolates were collected and stocked in the Asian Bacterial Bank (Seoul, Republic of Korea). Patients were excluded from the study if nasal MRSA carriage had been already known prior to the study period.

**Fig. 1:**
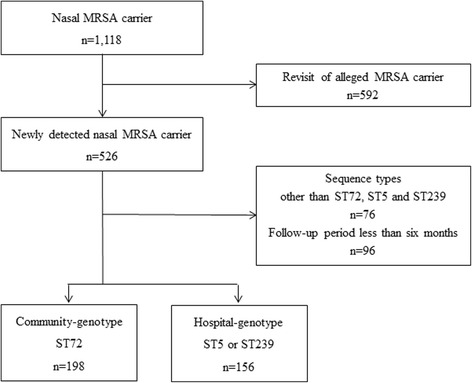
Flow chart summarizing identification of study cohorts

The patients carrying MRSA belonging to the sequence types other than ST72, ST5 and ST239 were excluded. We also excluded the patients who could not be followed up at least 6 months and had not developed subsequent MRSA infection until that time point (Fig. 1). Cohort 1 included the patients carrying MRSA with community-genotype, ST72, and cohort 2 included those who carried MRSA with hospital-genotypes, ST5 or ST239. If the patients developed MRSA infection multiple times, the first episode was only included in the analysis. This study was approved by the Institute Review Board of Samsung Medical Center.

### Data collection

Demographic and clinical characteristics, laboratory data, and outcome data were collected from the electronic medical records. The McCabe classification was used to categorize the severity of the underlying medical condition as non-fatal, ultimately fatal, or rapidly fatal [13]. Outcome variables included the rates of subsequent MRSA infection within six months after new detection of MRSA nasal colonization, overall mortality rates at six months, and time to development of subsequent MRSA infection. The type of infections was also described.

### Microbiological and molecular characterization

The Vitek 2 automated system (bioMerieux, Marcy l’Etoile, France) was used for microbiological identification and antimicrobial susceptibility testing. The MICs were determined according to the standards established by the Clinical and Laboratory Standards Institute (CLSI) [14]. Multilocus sequence typing (MLST) was carried out using PCR amplification and sequencing of seven housekeeping genes (*arcC, aroE, glpF, gmk, pta, tpi,* and *yqiL*), as previously described [15]. The allelic profiles and sequence types (STs) were assigned according to the MLST web site (http//saureus.mlst.net/).

### Statistical analysis

All tests of significance were two-tailed. *P*-values <0.05 were considered statistically significant. Categorical variables were compared between the groups using Chi-square test or Fisher’s exact test. Continuous variables were compared using the Mann–Whitney *U* test. Multivariate logistic regression analysis was used to determine the risk factors of subsequent MRSA infection. Stepwise model comparison was used to determine the best model using the PASW version 18.0 (SPSS Inc., Chicago, IL, USA). Times to development of subsequent MRSA infection during the follow-up period were calculated by the Kaplan-Meier method and compared between the cohorts using the log-rank test. Stata version 11.2 (StataCorp, College Station, TX, USA) was used for this analysis.

## Results

### Study population

We identified 1118 patients with nasal MRSA colonization during the study period. Among those patients, 592 known MRSA carriers revisiting the hospital were excluded. The patients carrying MRSA strains belonging to ST72, ST5 or ST239 who were followed up for at least 6 months were included in this study. A total of 354 patients carrying MRSA were included (Fig. 1). Among those MRSA isolates, the most frequent sequence type was ST72 (55.9%). ST5 and ST239 accounted for 23.8% and 5.4%, respectively. The most frequent reasons for nasal MRSA screening were transfer from other hospital (36.2%) and pre-operative screening (33.6%). Other reasons included MRSA isolation from clinical specimens (9.0%), carriage of VRE (6.5%), and undergoing hemodialysis or continuous ambulatory peritoneal dialysis (CAPD) (4.2%). Among the MRSA isolates from nasal carriers who were screened for pre-operative screening, ST72 (67.2%) was more frequent than ST5 or ST239 (Table 1).

**Table 1:** Comparison of demographic and clinical characteristics of the patients colonized with methicillin-resistant *Staphylococcus aureus* between ST72 and ST5 / ST239 cohorts

Variable	No. (%)		*P* value
	Community-genotype ST72 MRSA cohort (*n* = 198)	Hospital-genotype ST5 / ST239 MRSA cohort (*n* = 156)	
Female gender	77 (38.9)	61 (39.1)	0.967
Age, median (IQR)	53.0 (4.0–65.0)	65.5 (52.8–71.8)	0.177
Reason for nasal MRSA screening			
Transfer from other hospital	68 (34.3)	60 (38.5)	0.423
Pre-operative screening	80 (40.4)	39 (25.0)	0.002
MRSA isolated from clinical specimens	22 (11.1)	10 (6.4)	0.139
Carriage of vancomycin-resistant *Enterococcus*	10 (5.1)	13 (8.3)	0.213
Undergoing HD or CAPD	9 (4.5)	6 (3.8)	0.746
Location before admission			0.520
Tertiary hospital	141 (71.2)	113 (72.4)	
Home	34 (17.2)	26 (16.7)	
General hospital	19 (9.6)	11 (7.1)	
Chronic care facility	4 (2.0)	6 (3.8)	
Admission due to infection other than MRSA infection	32 (16.2)	39 (25.0)	0.039
Comorbidity			
Solid tumor	48 (24.2)	24 (15.4)	0.040
Cardiovascular disease	40 (20.2)	33 (21.2)	0.826
Diabetes mellitus	26 (13.1)	32 (20.5)	0.062
Liver disease	21 (10.6)	14 (9.0)	0.610
Neurologic disease	20 (10.1)	24 (15.4)	0.135
Chronic kidney disease	19 (9.6)	21 (13.5)	0.254
Solid organ transplantation	7 (3.5)	3 (1.9)	0.522
Chronic lung disease	5 (2.5)	10 (6.4)	0.072
Hematologic malignancy	4 (2.0)	11 (7.1)	0.031
CAPD	0 (0.0)	3 (1.9)	0.085
HSCT	0 (0.0)	1 (0.6)	0.441
Severity of underlying disease			0.651
Rapidly fatal	9 (4.6)	5 (3.2)	
Ultimately fatal	102 (51.5)	76 (48.7)	
Nonfatal	87 (43.9)	75 (48.7)	

*ST* sequence type, *IQR* Interquartile range, *HD* hemodialysis, *CAPD* Continuous ambulatory peritoneal dialysis, *HSCT* hematopoietic stem cell transplantation

### Comparison of characteristics of MRSA carriers between ST72 cohort and ST5 / ST239 cohort

The demographic and clinical characteristics of MRSA nasal carriers were compared between ST72 cohort and ST5 / ST239 cohort (Table 1). The most frequent comorbidity in ST72 cohort was solid tumor (24.2%), followed by cardiovascular disease, diabetes mellitus, liver disease, neurologic disease, and chronic kidney disease. Solid tumor was more frequent in ST72 cohort than in ST5 / ST239 cohort, whereas hematologic malignancy was more frequent in ST5 / ST239 cohort.

### Subsequent MRSA infection

**Fig. 2:**
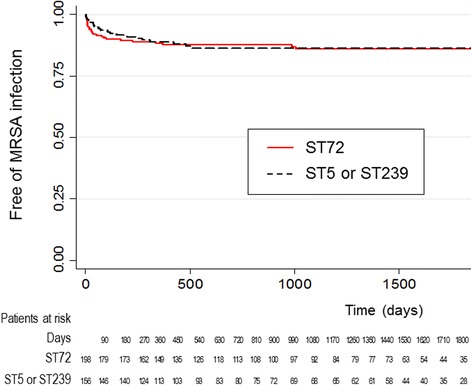
Kaplan-Meier curves for the cumulative risk of subsequent MRSA infection

The median duration of follow-up after detection of nasal MRSA carriage was 1058 days (range 11–2441 days) in ST72 cohort and 774.5 days (range 13–2515 days) in ST5 / ST239 cohort (*P* = 0.555). Among MRSA carriers of ST72 cohort, 13.1% developed subsequent MRSA infection similar to the rate in ST5 / ST239 cohort (12.8%) (*P* = 0.931). The median time to development of subsequent MRSA infection following new detection of nasal carriage was 27 days in ST72 cohort, which was not a statistically significant difference compared to ST5 / ST239 cohort (88 days). The Kaplan-Meier method was used to calculate the cumulative rate of being free of subsequent MRSA infection, and no significant difference was observed between two cohorts (*P* = 0.9209) (Fig. 2). Overall mortality rates did not differ between two cohorts (1.5% vs. 1.9%) (*P* = 1.000).

The most frequent type of subsequent MRSA infections was pneumonia (50%), followed by urinary tract infection (13.0%), primary bacteremia (10.9%), surgical site infection (8.7%), intra-abdominal infection (8.7%), and skin and soft tissue infection (6.5%). There was no difference in distribution of infection types between two cohorts. Among 26 cases of subsequent MRSA infections in ST72 cohort, 19 (73.1%) were hospital-onset.

### Risk factors for subsequent MRSA infection in MRSA nasal carrier cohorts

Multivariate analysis showed that the nasal carriers who also had MRSA isolated from culture of clinical specimens had 33-fold increased risk of subsequent MRSA infection (Table 2). Chronic lung disease and transfer from another hospital were also the risk factors for subsequent MRSA infection (O.R. 4.870 and O.R. 2.375, respectively).

**Table 2:** Risk factors for subsequent MRSA infection in MRSA nasal carrier cohorts

Variable	No. (%)		*P*	Adjusted OR (95% CI)	*P*
	Patients with subsequent MRSA infection (*n* = 46)	Patients with no subsequent MRSA infection (*n* = 308)			
Female gender	16 (34.8)	122 (39.6)	0.531		
Age, median (IQR)	57.5 (41.0–67.0)	52.5 (5.5–66.0)	0.111		
ST72 MRSA	26 (56.5)	172 (55.8)	0.931		
Reason for nasal MRSA screening					
Transfer from other hospital	24 (52.2)	104 (33.8)	0.015	2.375 (1.094–5.157	0.029
Pre-operative screening	11 (23.9)	108 (35.1)	0.135		
MRSA isolated from clinical specimens	23 (50.0)	9 (2.9)	<0.001	33.235 (13.102–84.303)	<0.001
Carriage of VRE	2 (4.3)	21 (6.8)	0.752		
Undergoing HD or CAPD	3 (6.5)	12 (3.9)	0.425		
Admission due to infection other than MRSA infection	17 (37.0)	54 (17.5)	0.002	0.576 (0.240–1.387)	0.219
Comorbidity					
Solid tumor	9 (16.9)	63 (20.5)	0.889		
Cardiovascular	10 (21.7)	63 (20.5)	0.841		
Diabetes mellitus	11 (23.9)	47 (15.3)	0.198		
Liver disease	6 (13.0)	29 (9.4)	0.442		
Neurologic disease	4 (8.7)	40 (13.0)	0.631		
Chronic kidney disease	8 (17.4)	32 (10.4)	0.162		
Solid organ transplantation	2 (4.3)	8 (2.6)	0.625		
Chronic lung disease	7 (15.2)	8 (2.6)	0.001	4.870 (1.193–19.872)	0.027
Hematologic malignancy	3 (6.5)	12 (3.9)	0.425		
CAPD	0 (0.0)	3 (1.0)	1.000		
HSCT	0 (0.0)	1 (0.3)	1.000		
Severity of underlying disease			0.335		
Rapidly fatal	3 (6.5)	11 (3.6)			
Ultimately fatal	26 (56.5)	152 (49.4)			
Nonfatal	17 (37.0)	145 (47.1)			

*MRSA* methicillin-resistant *Staphylococcus aureus*, *OR* odds ratio, *IQR* Interquartile range, *ST* sequence type, *VRE* vancomycin-resistant *Enterococcus*, *HD* hemodialysis, *CAPD* continuous ambulatory peritoneal dialysis, *HSCT* hematopoietic stem cell transplantation

### Predictive factors for ST72 MRSA nasal carriage

Multivariate analysis showed that predictive factors for ST72 MRSA among MRSA nasal carriage were pre-operative screening for MRSA nasal carriage (odds ratio 1.828; 95% CI 1.127–2.967; *P* = 0.015) and solid tumor (odds ratio 1.747; 95% CI 1.004–3.040; *P* = 0.048).

## Discussion

We found no evidence that rates of subsequent MRSA infection were different between community-genotype ST72 MRSA nasal carrier cohort and hospital-genotype ST5 / ST239 cohort. Comparison of the cumulative rates of being free of subsequent MRSA infection using the Kaplan-Meier method also revealed no significant difference between two cohorts. In most previous reports studying subsequent MRSA infection rates among MRSA carriers, the difference of infection rates according to the genotype was not studied. Our study is unique in this regard because we compared the subsequent MRSA infection rates between hospital-endemic MRSA clones and CA-MRSA clone highly prevalent in Korea.

A retrospective cohort study in the US tertiary care medical center showed that 33% of MRSA carriers developed MRSA infections in the following year [16]. Another retrospective cohort study in the US hospitals showed that persistent MRSA colonizers had an increased risk of death (HR 2.58) and MRSA infection (HR 10.89) [17]. A retrospective cohort study conducted in Alaska aiming to determine if carriage of CA-MRSA increased the risk for subsequent soft tissue infection revealed nasal MRSA carriage to be a significant risk factor for skin infections in the first year when compared with MSSA and non–*S. aureus* carriers [18]. In contrast, the study from the Asian countries is sparse. The report from the single center in Singapore showed that 14.4% of the MRSA carriers developed MRSA infection with median time to infection of 22 days [19].

It is interesting that ST72 MRSA cohort tended to have a shorter duration until developing subsequent MRSA infection compared to ST5 / ST239 cohort. A study from Singapore showed that risk of infection was much higher shortly after the initial acquisition; however the genotypes of MRSA were not determined [19]. In the retrospective cohort study from the US tertiary care medical center which also provided no information on genotypes showed that infection occurred a median of 56 days post-detection [16]. Higher virulence potential of CA-MRSA has been suggested in various community-genotype MRSA clones. USA300 clone is well known to be highly virulent [20]. Enhanced virulence of ST59 CA-MRSA over hospital-associated lineages was also demonstrated [21]. However, the virulence of ST72 MRSA needs to be more elucidated. Two studies comparing the virulence of ST72 MRSA with that of ST5 revealed lower virulence of ST72 MRSA strains [22, 23]. Several genes associated with adhesion and virulence were absent or rarely found in ST72 isolates.

Our study has demonstrated that isolation of MRSA from culture of clinical specimens was the most significant risk factor for subsequent MRSA infection in nasal carrier cohorts using the multivariate analysis. Although anterior nares are the most frequent carriage sites for *S. aureus*, multiple body sites can be colonized and CA-MRSA appears to be different in the distribution of colonization sites [7, 24]. As for USA300 strains, perirectal and inguinal area were common sites of colonization following the nares [25]. The frequencies of colonization in those areas were higher compared to non-USA300 strains.

There are some limitations in our study. First, this study focused on screening the nasal MRSA carriers according to the infection prevention and control policy of the hospital, and screening for the MRSA carriage at other body sites was not routinely performed. The MRSA surveillance study from the hospitals in Taiwan, in which majority of the isolates were clonotypes belonging to ST239, showed that nares cultures alone detected only 72.5% - 81.5% of all colonized patients [26]. As CA-MRSA strains are known to colonize various body sites rather than the nares frequently, the carriers who have a higher potential to develop subsequent MRSA infection could be excluded, affecting the results. Second, this was a retrospective design, and so many patients whose follow-up period less than 6 months were excluded from the analysis. It could affect the results. Third, the genotypes of MRSA isolates from subsequent infection were unknown, and so the relationship of MRSA isolates between the colonizing strains and the strains causing subsequent infection were not determined. Lastly, this is a single center study and caution should be exercised in generalizing our results.

## Conclusions

In conclusion, we found no evidence that rates of subsequent MRSA infection were different between newly-admitted patients carrying community-genotype ST72 MRSA and those whom carrying hospital-genotypes ST5 or ST239 MRSA in the following 6 months. This study also suggests the importance of post-discharge surveillance in determining the incidence of healthcare-associated infections due to MRSA, considering that infections occur after a considerable period of time after becoming a carrier.
